# Impact of Dupilumab on Skin Surface Lipid-RNA Profile in Severe Asthmatic Patients

**DOI:** 10.3390/cimb46100680

**Published:** 2024-10-15

**Authors:** Yoshihiko Sato, Hitoshi Sasano, Sumiko Abe, Yuuki Sandhu, Shoko Ueda, Sonoko Harada, Yuki Tanabe, Kyoko Shima, Tetsuya Kuwano, Yuya Uehara, Takayoshi Inoue, Ko Okumura, Kazuhisa Takahashi, Norihiro Harada

**Affiliations:** 1 Department of Respiratory Medicine, Juntendo University Faculty of Medicine and Graduate School of Medicine, Tokyo 113-8421, Japan; yo-sato@juntendo.ac.jp (Y.S.); h-sasano@juntendo.ac.jp (H.S.); su-abe@juntendo.ac.jp (S.A.); ysando@juntendo.ac.jp (Y.S.); sk-ueda@juntendo.ac.jp (S.U.); snharada@juntendo.ac.jp (S.H.); yutanabe@juntendo.ac.jp (Y.T.); kztakaha@juntendo.ac.jp (K.T.); 2 Research Institute for Diseases of Old Ages, Juntendo University Faculty of Medicine and Graduate School of Medicine, Tokyo 113-8421, Japan; 3 Atopy (Allergy) Research Center, Juntendo University Faculty of Medicine and Graduate School of Medicine, Tokyo 113-8421, Japan; kokumura@juntendo.ac.jp; 4 Biological Science Research, Kao Corporation, Tochigi 321-3426, Japan; shima.kyouko@kao.com (K.S.); kuwano.tetsuya@kao.com (T.K.); yyy.uehara@gmail.com (Y.U.); inoue.takayoshi@kao.com (T.I.)

**Keywords:** asthma, dupilumab, skin surface lipid, sebum, type 2 inflammation, epithelial barrier function

## Abstract

The analysis of skin surface lipid-RNAs (SSL-RNAs) provides a non-invasive method for understanding the molecular pathology of atopic dermatitis (AD), but its relevance to asthma remains uncertain. Although dupilumab, a biologic drug approved for both asthma and AD, has shown efficacy in improving symptoms for both conditions, its impact on SSL-RNAs is unclear. This study aimed to investigate the impact of dupilumab treatment on SSL-RNA profiles in patients with severe asthma. An SSL-RNA analysis was performed before and after administering dupilumab to asthma patients requiring this intervention. Skin samples were collected non-invasively from patients before and after one year of dupilumab treatment. Although 26 patients were enrolled, an SSL-RNA analysis was feasible in only 7 due to collection challenges. After dupilumab treatment, improvements were observed in asthma symptoms, exacerbation rates, and lung function parameters. Serum levels of total IgE and periostin decreased. The SSL-RNA analysis revealed the differential expression of 218 genes, indicating significant down-regulation of immune responses, particularly those associated with type 2 inflammation, suggesting potential improvement in epithelial barrier function. Dupilumab treatment may not only impact type 2 inflammation but also facilitate the normalization of the skin. Further studies are necessary to fully explore the potential of SSL-RNA analysis as a non-invasive biomarker for evaluating treatment response in asthma.

## 1. Introduction

The molecular composition of healthy skin relies on a delicate balance of various biomolecules that can be disrupted by diseases. Skin conditions such as cancer, psoriasis, and eczema, as well as internal organ disorders, can alter the molecular makeup of the skin [1]. Skin phenotypes encompass a range of components including lipids, proteins, inflammatory mediators, and immune cells, providing information on systemic conditions as well as skin health [1,2]. The skin, as the body’s outer surface, serves as a means of collecting biomolecules from samples such as sweat, hair, and the stratum corneum. Accordingly, it is a valuable source for monitoring both skin and body conditions. Previously, skin biopsy was necessary for RNA analysis due to the minimal RNA content in the stratum corneum. However, skin surface lipids (SSLs), collected using oil-blotting film, contain substantial human coding and non-coding RNAs [3]. Skin surface lipid-RNAs (SSL-RNAs), RNAs found within SSLs, primarily originate from the sebaceous glands, epidermis, and hair follicles. These RNAs are protected from degradation by RNases due to the surrounding lipids in SSLs. The non-invasive collection of SSL-RNAs can be achieved using a single oil-blotting film. AmpliSeq is a transcriptome method that targets the sequencing of more than 20,000 human transcripts and enables the detection of SSL-RNAs. These techniques allow for a deeper understanding of the physiological state of the skin [3]. The analysis of SSL-RNAs has emerged as a promising approach to understanding the pathophysiology of skin diseases, including atopic dermatitis (AD). Our previous studies have demonstrated the presence of human mRNAs in SSLs, suggesting their potential role in reflecting skin physiology and disease pathology [3]. Furthermore, an SSL-RNA analysis revealed significant alterations in gene expression patterns in patients with AD. The expression of *CCL17*, interleukin (IL)-1β (*IL1β*), *IL13*, S100 calcium-binding protein A9 (*S100A9*), and transforming growth factor β1 (*TGFβ1*) was significantly elevated in AD patients, while the expression of filaggrin (*FLG*) and involucrin (*IVL*) was significantly reduced compared to healthy controls. A gene ontology analysis was also observed in lipid synthesis-related genes in the sebaceous glands. This non-invasive method offers a valuable opportunity to explore the molecular pathology of skin diseases in pediatric patients without the need for invasive procedures such as biopsy.

Our study investigated the applicability of an SSL-RNA analysis to understand the molecular pathology of mild-to-moderate AD in children [4]. A comparison of sebum samples collected from healthy children and those with AD revealed differences in gene expression related to keratinization, lipid synthesis and storage, ceramide synthesis, antimicrobial peptides, and immune response. Furthermore, the expression levels of certain genes were correlated with the severity of AD, suggesting the potential utility of SSL-RNA analysis in evaluating disease severity and understanding the underlying molecular changes. Another study used an SSL-RNA analysis to investigate the molecular pathogenesis of very early onset AD in infants aged 1 and 2 months [5]. Infants with AD at 1 month of age showed altered gene expression patterns associated with lipid metabolism, antimicrobial peptides, immune responses, and barrier function. Interestingly, infants diagnosed with neonatal acne at 1 month who were finally diagnosed with AD at 2 months exhibited gene expression patterns similar to those with AD, indicating the predictive potential of SSL-RNA analysis to identify infants at risk of developing AD. These findings collectively underscore the utility of an SSL-RNA analysis as a non-invasive tool for elucidating the molecular mechanisms underlying AD and potentially predicting disease development in infants. Furthermore, we also conducted an SSL-RNA analysis in patients with Parkinson’s disease (PD) unrelated to skin disease and suggested the potential of SSL-RNA analysis to aid the early diagnosis of PD [6].

Asthma is one of the most common chronic diseases and is characterized by variable airflow limitation and hyperresponsiveness to bronchial air [7,8,9]. Among the various asthma phenotypes/endotypes, more than 80% of asthma patients are affected by type 2 inflammation [10,11,12]. ICSs effectively treat asthma with type 2 inflammation by inducing eosinophil apoptosis [13,14]. However, the impact of ICSs on asthma is limited due to steroid resistance in patients with the most severe clinical phenotype of asthma, resulting in refractory asthma. To address this issue, biologics with additional therapeutic benefits for intractable asthma have been developed, such as dupilumab, a humanized anti-IL-4 receptor α subunit monoclonal IgG4 antibody [15,16,17,18]. By blocking IL-4Rα, dupilumab acts as a dual antagonist against two related type 2 cytokines, IL-4 and IL-13. The biologic has shown clinical efficacy in various type 2 inflammations, including asthma and AD. An SSL-RNA analysis offers a non-invasive approach to comprehending the molecular pathology of AD, yet its applicability in asthma remains uncertain. In this study, we used SSL samples to comprehensively and non-invasively analyze SSL-RNAs in patients with severe asthma and aimed to investigate the effects of dupilumab treatment on SSL-RNAs in patients with severe asthma.

## 2. Materials and Methods

### 2.1. Study Participants

This study was conducted as a prospective non-interventional observational study. Enrolled were individuals diagnosed with severe asthma who were newly prescribed dupilumab between March 2019 and October 2022. The eligibility criteria were age ≥ 20 years and uncontrollable asthma symptoms and exacerbations that required oral corticosteroids despite receiving existing treatment options, including high-dose ICSs combined with long-acting β2 agonists and another controller medication. Patients who required dupilumab treatment as part of their insurance coverage for medical treatment were recruited from our outpatient clinic at Juntendo University Hospital (Tokyo, Japan). The diagnosis of asthma was established based on a clinical history characterized by episodic symptoms along with airflow limitation, as indicated by variations in lung function monitored using FEV_1_ or peak expiratory flow, in accordance with the guidelines outlined by the Global Initiative for Asthma [19]. Patients who met any of the following criteria were excluded from the study: (1) a diagnosis of eosinophilic granulomatosis with polyangiitis, interstitial pneumonia, infectious disease, or cancer; (2) current administration of other antibody preparations; (3) cases deemed inappropriate by the study investigators; (4) individuals receiving treatment with omalizumab, mepolizumab, and benralizumab within less than 1 month for the former two biologics and 2 months for the last dose of benralizumab, as well as those undergoing treatment with other biologics. The present study received approval from the Juntendo University Research Ethics Committee (Tokyo, Japan). Written informed consent was obtained from each patient prior to participation. The study was registered in the UMIN Clinical Trial Registry (UMIN000036302) on 27 March 2019 (Available online: http://www.umin.ac.jp/ (accessed on 2 April 2024)).

Physiological evaluations were performed at the following time points: initial administration and 1 year after administration relative to the initial administration of dupilumab. The evaluations included the asthma control test (ACT), pulmonary function tests, oscillometry (also known as the forced oscillation technique), measurement of FeNO levels and blood sampling. FeNO levels were evaluated according to the recommendations of the American Thoracic Society. Measurements were taken at a constant flow rate of 0.05 L/s against an expiratory resistance of 20 cm of water using an electrochemical handheld NO analyzer (NIOX VERO^®^; Aerocrine AB, Solna, Sweden).

### 2.2. Classification According to Response

Patients were classified as responders or non-responders based on their response to dupilumab treatment, and considered changes in ACT score, lung function, and asthma exacerbations, with reference to previous studies [20,21,22,23,24,25,26]. Classification as a responder was based on meeting at least two of the following three criteria after 1 year of dupilumab treatment without significant deterioration in any other criterion: (1) an improvement in the ACT score by at least 3 points (including patients who achieved an ACT score of 25 points), which was previously suggested as the minimal clinically important difference [27,28]; (2) a reduction in the number of asthma exacerbations, including patients who had no exacerbations before and after treatment; (3) an improvement in FEV1 of at least 100 mL [26,29]. Significant deterioration was defined based on the following criteria: (a) a decrease in the ACT score of at least 3 points, (b) an increase in the number of exacerbations, and (c) a decrease in FEV1 of at least 100 mL.

### 2.3. Quantification of Circulating Lymphocyte Frequency

A flow cytometry analysis was performed following the previously described protocol [30,31]. Briefly, peripheral venous blood samples were collected in tubes containing heparin, and PBMCs were purified by density gradient centrifugation using Ficoll–Paque Plus solution (Cytiva, Tokyo, Japan) at a concentration of 3 × 10^6^ cells per well. The cells were then stained with various combinations of suitable antibodies for 30 min at 4 °C. In this study, the following surface marker antibodies were used: anti-CD3-APC-H7, anti-CD4-FITC, anti-CD19-FITC, anti-CD56-PE-CF594, anti-CD117 (c-Kit)-PE-CF594 (BD Biosciences, San Jose, CA, USA), anti-BDCA2-FITC, anti-CD1a-FITC, anti-CD3-FITC, anti-CD11c-FITC, anti-CD14-FITC, anti-CD25-PE, anti-CD34-FITC, anti-CD123-FITC, anti-CD127 (IL-7Rα)-Brilliant Violet 605, anti-CD127 (IL-7Rα)-Brilliant Violet 421, anti-CD161-PerCPCy5.5, anti-CD183 (CXCR3)-APC, anti-CD194 (CCR4)-Brilliant Violet 510, anti-CD196 (CCR6)-PerCPCy5.5, anti-CD294 (CRTH2)-Brilliant Violet 421, anti-CD294 (CRTH2)-Brilliant Violet 510, and anti-FCɛR1-FITC. Negative lineage markers (Lin^−^) were defined as CD1a^−^, CD3^−^, CD11c^−^, CD14^−^, CD19^−^, CD34^−^, TCRγδ^−^, CD123^−^, BDCA2^−^, and FCɛR1^−^. Th1 cells were identified as CD3^+^, CD4^+^, CCR6^−^, and CXCR3^+^ cells; Th2 cells as CD3^+^, CD4^+^, CCR6^−^, CXCR3^−^, and CRTH2^+^ cells; Th17 cells as CD3^+^, CD4^+^, CCR6^+^, and CXCR3^−^ cells; Tregs as CD3^+^, CD4^+^, CD25^+^, and CD127^−^ cells; natural killer (NK) cells as CD3^−^ and CD56^+^ cells; ILC1 as Lin^−^, CD127^+^, CD161^+^, CD117^−^, and CRTH2^−^ cells; ILC2 as Lin^−^, CD127^+^, CD161^+^, and CRTH2^+^ cells; and ILC3 as Lin^−^, CD127^+^, CD161^+^, CD117^+^, and CRTH2^−^ cells. Dead cells were identified using the Zombie Fixable Viability Kit (BioLegend, San Diego, CA, USA), followed by doublet exclusion in forward scatter and side scatter. After overnight fixation, cells were analyzed using a fluorescence-activated cell sorting (FACS) LSRFortessa cell analyzer (BD Biosciences, Franklin Lakes, NJ, USA). The FlowJo software program (version 10; BD Biosciences) was used to evaluate the FACS data.

### 2.4. Quantification of Serum Periostin Levels

After centrifugation of the density gradient of the blood samples, the sera of the patients were collected and frozen at −80 °C. Following a standardized protocol, serum periostin levels were measured using ELISA (Shino-Test, Kanagawa, Japan) as previously described [32]. The working range of the assay for periostin ranged from 0 (lower limit of quantification) to 2000 (upper limit of quantification).

### 2.5. Collection of SSLs, RNA Preparation, and AmpliSeq Transcriptomic Analysis

SSLs were collected from participants’ faces, following established protocols [3,4]. In summary, sebum samples were obtained by gently wiping the skin surface with a single sheet of oil-blotting film (5.0 cm × 8.0 cm; 3M Japan, Tokyo, Japan) per participant. RNA extraction was performed using QIAzol reagent (QIAGEN, Valencia, CA, USA) from the oil-blotting film, followed by purification using the RNeasy Mini Kit (QIAGEN). Sequence library preparation, template preparation, and sequencing were carried out using the Ion AmpliSeq Transcriptome Human Gene Expression Kit, Ion Chef System, and Ion S5 XL System (Thermo Fisher Scientific, Waltham, MA, USA).

### 2.6. Statistical Analysis

Data analyses were performed as described previously [3,4]. Normalization and statistical analysis of the AmpliSeq transcriptomic data were performed using the R language. The Ion Torrent Suite software version 5.16 (Thermo Fisher Scientific) was used to obtain the read count data. The package R DESeq2 (Bioconductor) 25 was used for normalization and quality control of the read count data. We removed from the analysis samples in which the proportion of genes detected among the 20,802 target genes analyzed in the Ion AmpliSeq Transcriptome Human Gene Expression Kit was less than 20%. Only genes for which one or more reads were detected in ≥90% of the samples were normalized by the size factor.

Differentially expressed genes (DEGs) between 0 months (pre-treatment) and 12 months (after 12 months of treatment) were analyzed using the likelihood ratio test, and those with a false discovery rate (FDR) were adjusted using the Benjamini–Hochberg method below the threshold value of 0.05. A gene ontology (GO) analysis of genes obtained by differential expression analysis was performed using Reactome (Available online: https://reactome.org/ (accessed on 26 May 2024)). To evaluate the eosinophil-related, Th2-related, and skin barrier-related gene signatures, a gene set variation analysis (GSVA) was performed using various previously reported gene sets [33,34,35,36,37]. Enrichment scores for each gene set at 0 months (pre-treatment) and 12 months (after 12 months treatment) were calculated. Comparisons between 0 months (before treatment) and 12 months (after 12 months of treatment) were made using paired *t*-tests.

## 3. Results

### 3.1. Participant Characteristics

Twenty-six patients met the study criteria and treated with dupilumab, and sebum samples were collected. An SSL-RNA analysis before and after dupilumab administration was performed in seven of the patients (four males, three females). Insufficient sebum could be collected for RNA analysis in the remaining 19 patients (4 males, 15 females), probably due to face washing or makeup. Table 1 lists the clinical characteristics of the study participants. The median age was 47 years (range: 30–71 years). Two patients were ex-smokers and five were non-smokers. One patient was receiving a regular prednisolone regimen of 1 mg/day that was prescribed by a collagen disease specialist for fibromyalgia management rather than asthma. The median daily dose of ICSs was 1000 µg. Median duration of asthma was 14 years (range, 2–37 years). One patient switched from omalizumab treatment, and three patients switched from benralizumab. The remaining three patients had not previously received biologics treatment. Four patients had AD and three had chronic sinusitis. Median FEV_1_% was 80.5% and median peripheral eosinophil count was 92/μL. Four patients were classified as responders.

### 3.2. Change in Each Parameter 1 Year after Dupilumab Treatment

After one year of dupilumab treatment, four of the seven patients had an improvement in ACT score of at least 3 points, met the threshold for minimal clinically important difference (MCID), or achieved total control (Table 2 and Supplementary Figure S1) [27,28]. The number of asthma exacerbations significantly decreased and peak expiratory flow rate significantly increased (Table 2 and Supplementary Figure S1). Four patients were responders and three were non-responders (Table 2). Although there appeared to be no distinct relationship between response to dupilumab treatment and patient characteristics such as comorbidities, eosinophil count, and airflow limitation, all three patients who had not previously received biologic treatment were responders (Table 1 and Table 2). One non-responder had a deterioration in airflow limitation despite treatment with dupilumab (Table 2). Peripheral blood eosinophils increased after dupilumab treatment in four patients, three of whom had switched from benralizumab treatment. Additionally, peripheral blood lymphocytes significantly increased, and serum levels of total IgE and periostin significantly decreased in all seven patients after treatment (Table 2 and Supplementary Figure S1).

The gating strategy used for analysis of the frequency of PBMCs in peripheral blood using flow cytometry is illustrated in Supplementary Figure S2. We assessed the prevalence of Th cells and ILCs by comparing their numbers to CD3^+^ and CD4^+^ cells, and to Lin^−^ CD127^+^ and CD161^+^ cells, respectively, and we represented the frequency of natural killer (NK) cells in relation to lymphocytes. Although there was variation among the patients, there was a significant increase in ILC3s in peripheral blood lymphocyte fraction after 1 year (Table 2 and Supplementary Figure S1).

### 3.3. Differential Expression Analysis Using SSL-RNAs in Patients with Severe Asthma before and after 1 Year of Dupilumab Treatment

**Figure 1:**
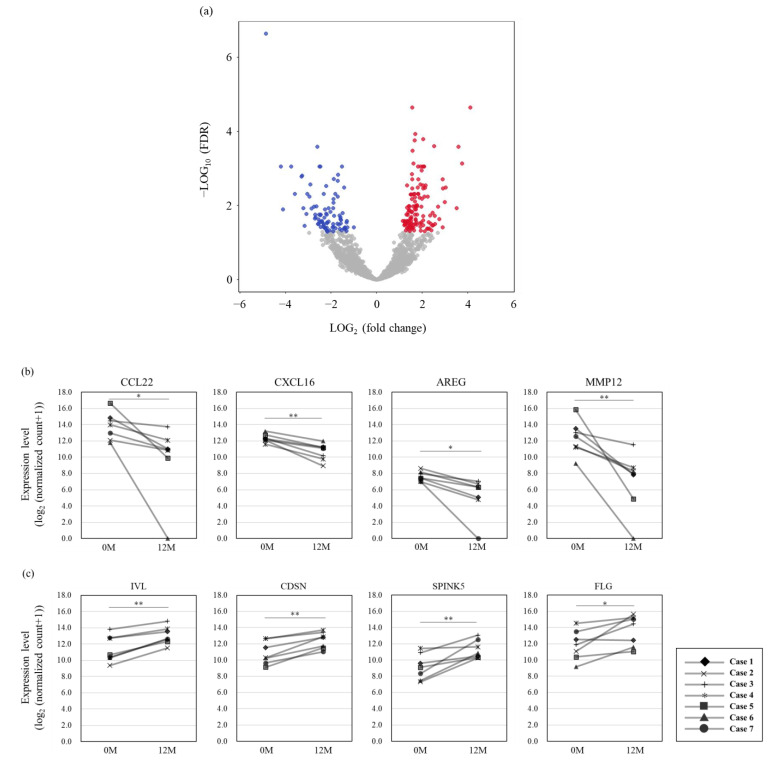
(**a**) Volcano plot of differentially expressed genes between 12 months of dupilumab treatment and 0 months (pre-treatment). The red dots indicate genes up-regulated 12 months after dupilumab treatment and the blue dots indicate those down-regulated at 12 months (Benjamini and Hochberg’s false discovery rate (FDR)  <  0.05). Change in expression level of inflammation (**b**) and skin barrier-related genes (**c**) between 0 months and 12 months after dupilumab treatment. (** FDR < 0.01, * FDR < 0.05; Benjamini and Hochberg’s false discovery rate (FDR)  <  0.05).

**Figure 2:**
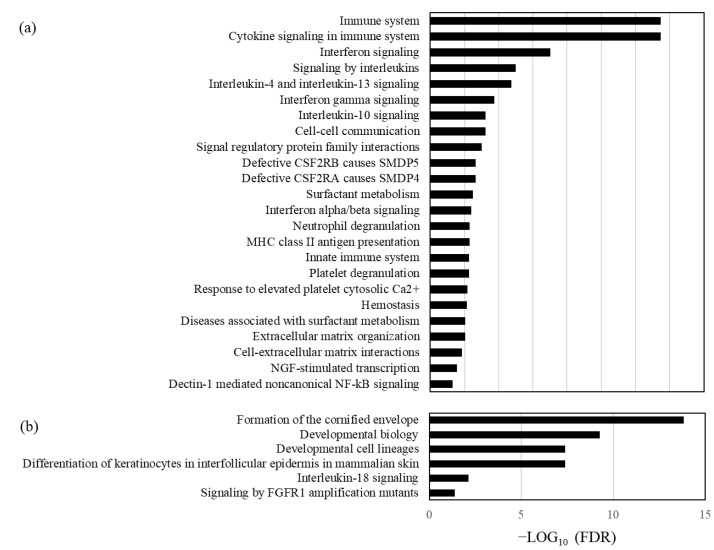
Results of enrichment analysis for the Reactome database in down-regulated genes (**a**) and up-regulated genes (**b**) 12 months after dupilumab treatment (Benjamini and Hochberg’s false discovery rate (FDR) < 0.05).

**Figure 3:**
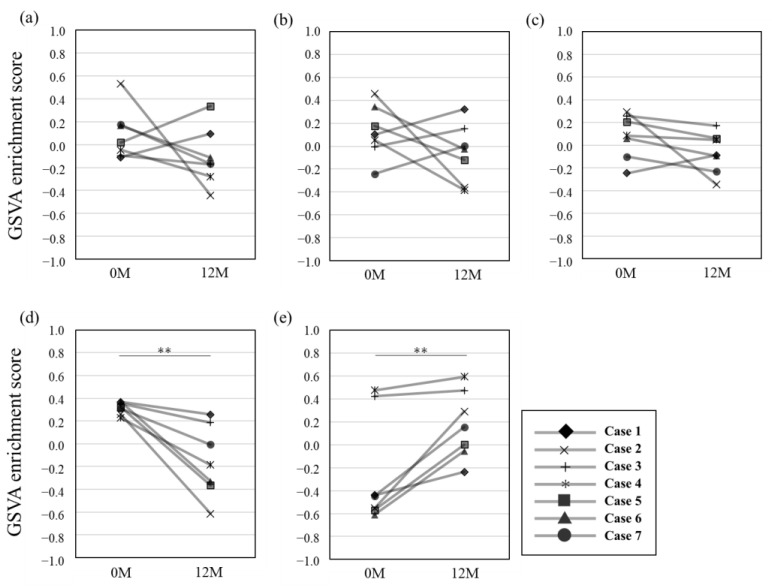
Results of GSVA. Change in score of the three eosinophil-related (#1, #2, #3; (**a**), (**b**), (**c**), respectively), Th2-related (**d**), and skin barrier-related (**e**) gene signatures between 0 months and 12 months after dupilumab treatment. GSVA: gene set variation analysis. #1, #2, #3; GSVA signature scores of the three eosinophil-related gene set. ** *p* < 0.01; paired *t*-test.

In differential expression analysis conducted to understand the biological characteristics of SSL-RNA profiles in severe asthma patients undergoing 1 year of dupilumab treatment, a comparison of patient profiles before and after treatment revealed 218 differentially expressed genes (DEGs), with 128 genes up-regulated and 90 genes down-regulated after treatment (Figure 1, details in Supplementary Table S1). The differentially expressed genes included those related to inflammation (*CCL22*, *CXCL16*, *AREG*, *MMP12*) involved in asthma pathophysiology, as well as genes associated with the skin barrier (*IVL*, *CDSN*, *SPINK5*, *FLG*) (Figure 1). A GO analysis using Reactome (Available online: https://reactome.org/ (accessed on 26 May 2024)) highlighted significant down-regulation of the immune system, cytokine signaling in the immune system, IL-4 and IL-13 signaling, and interferon signaling, along with up-regulation of cornified envelope formation, differentiation of keratinocytes in the interfollicular epidermis in mammalian skin and IL-18 signaling (Figure 2). A GSVA was used to calculate an enrichment score, using eosinophils and the Th2-related gene signature from previous reports to assess immune-related changes after dupilumab treatment. At the one-year mark, although no alterations were observed in eosinophil-related signatures, scores for Th2 immune response-related genes decreased significantly, and scores for barrier-related genes increased significantly (Figure 3).

## 4. Discussion

In a small subset of the present asthmatic patients, dupilumab treatment demonstrated improvements in airflow limitation and reductions in serum levels of total IgE and periostin. There was also a shift in the frequency of the ILC1 and ILC3 subsets in peripheral blood. This study is the first to investigate SSL-RNA dynamics before and after dupilumab treatment in patients with severe asthma. After one year of treatment, the differential expression analysis revealed 90 down-regulated DEGs, including crucial genes involved downstream of type 2 inflammation such as *TGFB1* and *AREG*. The GO analysis and GSVA revealed a notable down-regulation of immune-related processes, particularly genes associated with the Th2 immune response and IL-4 and IL-13. Furthermore, there was an up-regulation of genes related to the formation of the cornified envelope, keratinization, and barrier function. These findings suggest that one year of dupilumab treatment may attenuate immune responses and promote improved skin health. Treatment with dupilumab led to the down-regulation of genes associated with Th2 immunity and type 2 cytokines such as IL-4 and IL-13, as well as genes encoding proteins like TGF-β1 and amphiregulin, which are involved in downstream type 2 inflammation. The reduction in these type 2 inflammation-related genes, along with the decrease in serum periostin levels, suggests a possible association with improved airway remodeling. However, despite these changes, improvements in airflow restriction and reductions in peripheral blood Th2 cells and ILC2 were not clearly observed. These discrepancies indicate that the results of the SSL-RNA analysis may reflect the local conditions of the skin, but not necessarily those of the peripheral blood or airways. The present differential expression and GO analyses suggest an up-regulation of IL-18 signaling. In addition to its involvement in Th2 cell development and IgE production, IL-18 plays a crucial role in Th1 cell differentiation [38,39]. In particular, IL-18 is also associated with asthma [38,39]. It is plausible that whereas dupilumab suppresses type 2 inflammation, a feedback mechanism could persist to maintain type 2 inflammation through pyroptosis and IL-18 production. Further research is required to elucidate this feedback mechanism. In addition, three of the seven patients did not have atopic dermatitis as a comorbidity, suggesting that their skin did not have local type 2 inflammation before dupilumab treatment. It is important to emphasize that all validated patients in this study received dupilumab treatment, which affects cutaneous RNA. It should be noted that evaluating both the condition of the airways and the skin requires careful examination and is not easily determined. The present findings suggest a reduction in type 2 immune responses with dupilumab. However, additional case collection and research is necessary to determine whether this change in SSL-RNAs indicates only a reduction in type 2 inflammation in the skin or if it also reflects type 2 inflammation in asthma.

Although dupilumab treatment led to significant changes in peripheral blood eosinophils, no eosinophil-related alterations were observed in SSL-RNAs. This discrepancy could be due to several factors, including the low proportion of eosinophils in the sebum, which may not be adequately represented by SSL-RNAs. It could also be attributed to the lack of altered eosinophil levels at the skin site or variations in peripheral blood eosinophils, which vary greatly between individuals.

The primary limitation of this study is its exceptionally small sample size. Despite our instructions to minimize face washing and makeup application before sebum collection, the high rate of unsuccessful SSL-RNA collection among females (15/18, 83%) suggests the potential influence of face washing and makeup on results. In addition, sample collection was unsuccessful in 4/8 (50%) of the male participants. These participants visiting our hospital in central Tokyo may have faced difficulties in refraining from washing their faces and applying makeup prior to sebum collection. Although sebum collection is non-invasive and an excellent candidate for biomarkers, the collection method needs to be further investigated. Additionally, more studies are needed to determine whether information obtained from local sebum can apply to the entire body or local airways. Another major limitation was that of the seven patients, four had a predicted FEV_1_ > 100% and four could have affected peripheral blood eosinophil or lymphocyte fractions influenced by biologic treatments. However, it is noteworthy that even against this patient background, SSL-RNAs were shown to reduce type 2 immune responses and improve epithelial barrier function. In addition, a principal component analysis of the SSL-RNA profiles did not reveal any notable differences between the female and male groups. However, the small number of samples analyzed makes it difficult to conclude on the influence of sex differences.

## 5. Conclusions

This study is the first report to demonstrate that SSL-RNAs from asthmatic patients treated with dupilumab showed a reduction in immune responses, particularly genes related to type 2 inflammation, and a potential improvement in epithelial barrier function. These findings suggest that dupilumab, which targets IL-4 and IL-13, may not only impact type 2 inflammation but also promote the normalization of the skin. Further research is needed to evaluate the potential use of non-invasive SSL-RNA analysis as a biomarker.

## Figures and Tables

**Table 1:** Baseline characteristics.

	Case 1	Case 2	Case 3	Case 4	Case 5	Case 6	Case 7	Median
Sex (M/F)	F	M	M	M	F	M	F	NA
Age (y)	47	69	41	57	30	71	42	47.0
Age at asthma onset (y)	33	57	6	20	3	60	40	33.0
Duration of asthma (y)	14	12	35	37	27	11	2	14.0
BMI (kg/m^2^)	27.7	20.8	26.0	22.2	25.7	27.3	19.0	25.7
Smoking history (pack years)	0	10	0	0	0	40	0	0.0
Allergic rhinitis	+	+	+	–	+	+	+	NA
Atopic dermatitis	+	+	+	–	+	–	–	NA
Chronic sinusitis	–	+	–	+	+	–	–	NA
NERD	–	+	–	–	+	–	–	NA
Daily dose of ICSs (FP equivalent dose, µg)	1440	1000	1000	1000	1000	1000	1000	1000
Daily dose of OCSs (PSL equivalent dose, mg)	1	0	0	0	0	0	0	0.0
Biologics before switching	Benralizumab	Benralizumab	–	–	Omalizumab	Benralizumab	–	NA
Asthma exacerbations (/year)	1	5	2	1	7	5	12	5.0
Unscheduled visits (/year)	0	2	2	0	4	0	3	2.0
Hospitalizations (/year)	0	0	0	0	0	0	0	0.0
ACT score points	23	24	16	10	16	19	10	16.0
FeNO (ppb)	56	126	34	30	21	11	15	30.0
FVC (L)	3.1	3.2	5.3	2.9	3.1	2.5	3.2	3.1
%FVC (predicted, %)	116.0	84.1	112.0	76.7	96.0	71.1	104.2	96.0
FEV_1_ (L)	2.5	1.7	4.4	0.8	2.9	2.0	2.8	2.5
%FEV_1_ (predicted, %)	109.2	54.8	107.4	26.2	101.3	68.4	108.8	101.3
FEV_1_% (%)	80.5	53.1	83.1	28.7	92.0	78.0	88.9	80.5
PEFR (L/s)	6.9	5.7	12.4	3.1	8.5	8.5	6.2	6.9
Peripheral neutrophils (×10^2^ cells/μL)	32.0	48.8	61.0	90.0	48.0	47.0	67.0	48.8
Peripheral eosinophils (cells/μL)	0.0	0.0	92.0	684.0	101.0	0.0	138.0	92.0
Peripheral basophils (cells/μL)	0.0	7.1	561.2	63.0	28.8	0.0	51.6	28.8
Peripheral lymphocytes (×10^2^ cells/μL)	10.2	18.1	13.2	11.8	18.4	12.5	13.2	13.2
Total IgE (IU/mL)	4245	217	1361	2168	123	8	15	217.0
Periostin (ng/mL)	54.9	325.7	33.9	83.3	40.8	48.5	52.4	52.4
Th1 cells (% of Th cells, %)	18.9	24.8	22.8	19.9	9.1	23.1	25.6	22.8
Th2 cells (% of Th cells, %)	75.1	70.6	74.1	68.4	83.5	68.5	62.7	70.6
Th17 cells (% of Th cells, %)	1.9	1.5	1.1	3.6	3.2	1.5	2.9	1.9
Treg cells (% of Th cells, %)	5.2	5.5	9.6	6.3	4.5	3.2	4.4	5.2
ILC1 (% of ILC cells, %)	85.9	72.8	65.5	62.5	50.1	45.6	52.8	62.5
ILC2 (% of ILC cells, %)	8.1	18.4	28.7	32.0	14.8	25.6	34.5	25.6
ILC3 (% of ILC cells, %)	6.0	8.2	5.8	5.5	35.1	28.9	12.7	8.2
NK cells (% of lymphoid cells, %)	4.7	19.9	12.8	19.2	5.7	25.2	20.6	19.2

Abbreviations for tables: ACT, asthma control test; BMI, body mass index; FeNO, fractional exhaled nitric oxide; FEV_1_, forced expiratory volume in 1 s; FEV_1_%, FEV_1_ second/forced vital capacity; FP, fluticasone propionate; FVC, forced vital capacity; ICSs, inhaled corticosteroids; IgE, immunoglobulin E; ILC, innate lymphoid cell; NA, not applicable; NERD, nonsteroidal anti-inflammatory drug-exacerbated respiratory disease; NK, natural killer; OCSs, oral corticosteroids; PEFR, peak expiratory flow rate; PSL, prednisolone; Th, helper T; Treg, regulatory T.

**Table 2:** Change in parameters before and after 1 year of dupilumab treatment *.

	Case 1	Case 2	Case 3	Case 4	Case 5	Case 6	Case 7	Median
BMI (kg/m^2^)	27.3 (0)	25.8 (5)	26.8 (1)	27.6 (5)	27.9 (2)	26.1 (−1)	17.2 (−2)	27 (0.8)
Asthma exacerbations (/year)	0 (−1)	0 (−5)	0 (−2)	0 (−1)	3 (−4)	3 (−2)	3 (−9)	0 (−2)
Unscheduled visits (/year)	0 (0)	0 (−2)	0 (−2)	0 (0)	2 (−2)	1 (1)	1 (−2)	0 (−2)
Hospitalizations (/year)	0 (0)	0 (0)	0 (0)	0 (0)	0 (0)	0 (0)	0 (0)	0 (0)
ACT score	24 (1)	25 (1)	25 (9)	18 (8)	14 (−2)	20 (1)	19 (9)	20 (1)
Responder/non-responder	non-responder	responder	responder	responder	non-responder	non-responder	responder	NA
FeNO (ppb)	18 (−38)	33 (−93)	33 (−1)	64 (34)	23 (2)	12 (1)	12 (−3)	23 (−1)
FVC (L)	3.0 (−0.1)	3.7 (0.5)	5.3 (0.1)	3.2 (0.2)	3.1 (−0.0)	2.7 (0.1)	3.4 (0.2)	3.2 (0.1)
%FVC (predicted, %)	114.1 (−1.9)	97.8 (13.7)	112.5 (0.5)	83.5 (6.8)	94.2 (−1.8)	75.4 (4.3)	112.4 (8.2)	97.8 (4.3)
FEV_1_ (L)	2.5 (0.0)	2.3 (0.6)	4.4 (0.0)	1.9 (1.0)	2.8 (−0.1)	2.1 (0.1)	3.0 (0.2)	82.6 (0.1)
%FEV_1_ (predicted, %)	113 (3.8)	75.8 (21)	107.4 (0)	59.1 (32.9)	98.9 (−2.4)	72.4 (4)	116.8 (8)	98.9 (4)
FEV_1_% (%)	84.6 (4.1)	63.0 (9.9)	82.6 (−0.5)	59.2 (30.5)	91.2 (−0.8)	77.5 (−0.5)	88.2 (−0.7)	82.6 (−0.5)
PEFR (L/s)	6.9 (−0.1)	9.3 (3.6)	13.7 (1.3)	5.0 (1.9)	8.8 (0.3)	8.9 (0.5)	7.1 (0.9)	8.8 (0.9)
Peripheral neutrophils (×10^2^ cells/μL)	24.6 (−7.4)	55.8 (7.0)	31.8 (−29.2)	36.8 (−53.2)	39.8 (−8.2)	54.8 (7.83)	51.6 (−15.4)	39.8 (−8.2)
Peripheral eosinophils (cells/μL)	62 (62)	1166 (1166)	52 (−40)	1030 (346)	78 (−23)	40 (40)	70 (−68)	70.0 (40)
Peripheral basophils (cells/μL)	11.7 (11.7)	39.2 (32.1)	40.6 (−520.6)	50.4 (−12.6)	19.5 (−9.3)	23.7 (23.7)	49 (−2.6)	39.2 (−2.6)
Peripheral lymphocytes (×10^2^ cells/μL)	11.1 (0.9)	25 (6.9)	20.4 (7.2)	19.8 (8)	18.8 (0.4)	16.4 (3.8)	13.7 (0.5)	18.8 (3.8)
Total IgE (IU/mL)	1346 (−2899)	6 (−211)	369 (−992)	202 (−1966)	20 (−103)	4 (−4)	17 (2)	20 (−211)
Periostin (ng/mL)	48 (−6.9)	68.4 (−257.2)	33.4 (−0.5)	58.8 (−24.5)	42.9 (2.1)	42.2 (−6.2)	49.2 (−3.2)	48 (−6.2)
Th1 cells (% of Th cells, %)	17.7 (−1.2)	22.4 (−2.4)	19.6 (−3.2)	21.5 (1.6)	8.7 (−0.5)	15.3 (−7.8)	21.7 (−3.9)	19.6 (−2.4)
Th2 cells (% of Th cells, %)	74.6 (−0.5)	69.4 (−1.2)	74.9 (0.8)	69.6 (1.2)	87.2 (3.7)	80.3 (11.8)	69.4 (6.7)	74.6 (1.2)
Th17 cells (% of Th cells, %)	2.2 (0.4)	4.3 (2.9)	1.8 (0.7)	2.4 (−1.3)	1.6 (−1.6)	1.5 (0.1)	2.3 (−0.6)	2.2 (0.1)
Treg cells (% of Th cells, %)	3.8 (−1.4)	4.6 (−0.9)	10.5 (0.9)	5.8 (−0.6)	4.4 (−0.1)	3.9 (0.7)	5.7 (1.3)	4.6 (−0.1)
ILC1 (% of ILC cells, %)	73.5 (−12.4)	45.7 (−27.1)	39 (−26.5)	59.6 (−2.9)	40.3 (−9.8)	29.3 (−16.3)	66 (13.2)	45.7 (−12.4)
ILC2 (% of ILC cells, %)	8.1 (0)	28.9 (10.5)	42.2 (13.5)	28 (−4)	22.6 (7.8)	44.3 (18.7)	16.4 (−18.1)	28 (7.8)
ILC3 (% of ILC cells, %)	18.7 (12.8)	24.8 (16.6)	18.7 (12.9)	12.4 (6.9)	37.2 (2.1)	26.4 (−2.5)	17.7 (5)	18.7 (6.9)
NK cells (% of lymphoid cells, %)	17 (12.6)	24 (4.1)	16.5 (3.7)	26.9 (7.7)	11.2 (5.5)	20.4 (−4.8)	20.4 (-0.2)	20.4 (4.1)

* The values in this Table represent data collected after one year of treatment, with the figures in parentheses indicating the difference between the one-year and baseline values. Abbreviations for tables: ACT, asthma control test; BMI, body mass index; FeNO, fractional exhaled nitric oxide; FEV_1_, forced expiratory volume in 1 s; FEV_1_%, FEV_1_ second/forced vital capacity; FVC, forced vital capacity; IgE, immunoglobulin E; ILC, innate lymphoid cell; NA, not applicable, NK, natural killer; PEFR, peak expiratory flow rate; Th, helper T; Treg, regulatory T.

## Data Availability

The original contributions presented in the study are included in the article/Supplementary Materials, further inquiries can be directed to the corresponding author/s.

## Supplementary Materials

The following supporting information can be downloaded at: https://www.mdpi.com/article/10.3390/cimb46100680/s1, Supplementary Figure S1: Changes in parameters from baseline to 1 year after dupilumab treatment; Supplementary Figure S2: The gating strategy for the PBMCs; Supplementary Table S1: Differentially expressed genes between 12 months of dupilumab treatment and 0 months (pre-treatment).

## Abbreviations

ACT: asthma control test; FEV_1_%, FEV_1_ second/forced vital capacity; FP, fluticasone propionate; FVC, forced vital capacity; NA, not applicable; NERD, non-steroidal anti-inflammatory drug-exacerbated respiratory disease; NK, natural killer; PSL, prednisolone.
