# Supplementary figures and images for: Impact of Dupilumab on Skin Surface Lipid-RNA Profile in Severe Asthmatic Patients
Source: Curr Issues Mol Biol. 2024 Oct 15;46(10):11425–37. doi: 10.3390/cimb46100680 (PMC11505614; doi:10.3390/cimb46100680)

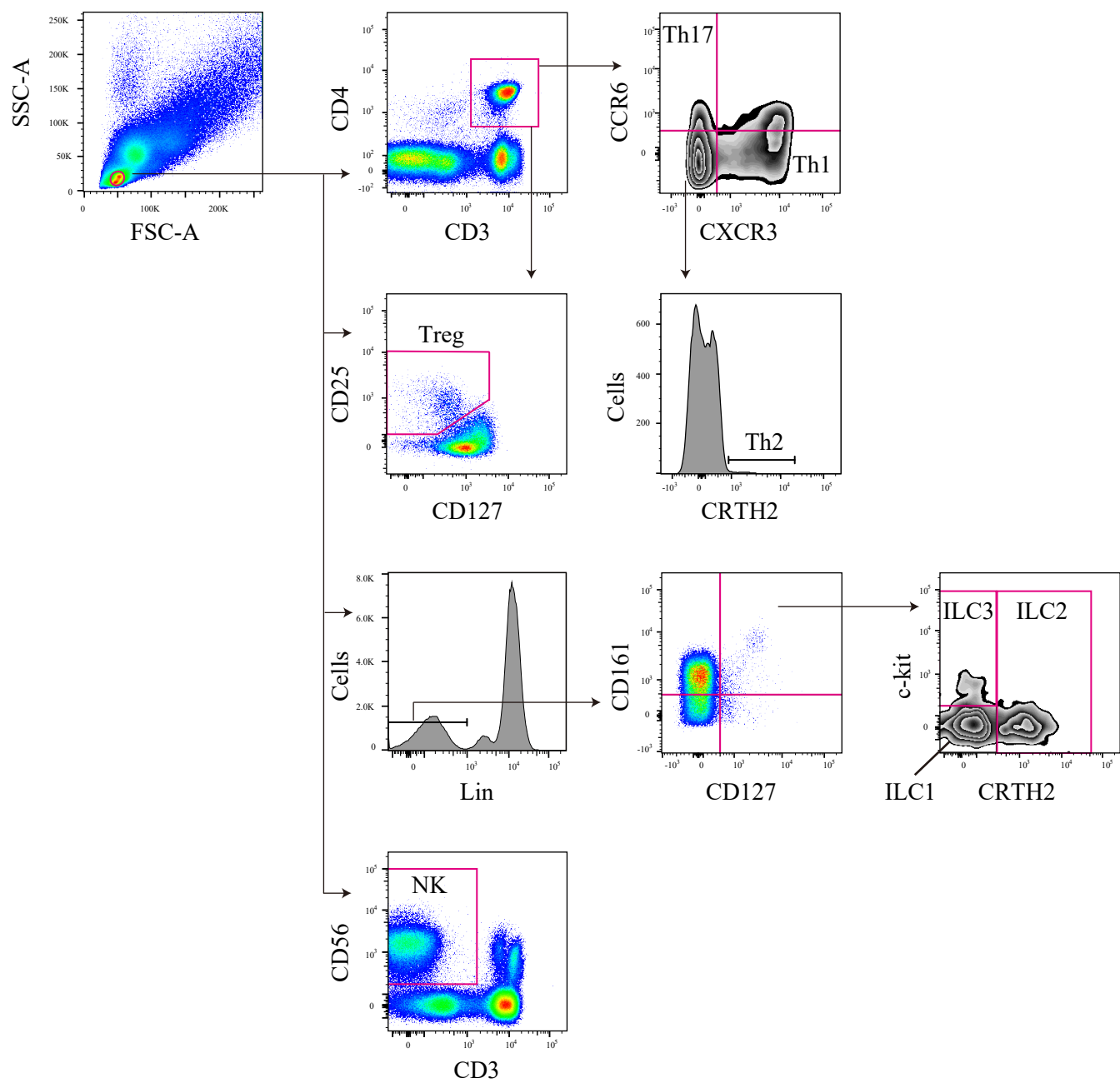

**Supplementary Figure S2.** The gating strategy for the PBMCs.

Supplement: Supplementary file 1 [file cimb-46-00680-s001.zip › cimb-3216055 R2 Supplementary Figure S2.pdf]
